# Hepatocellular-Cholestatic Pattern of Liver Injury in a Patient With Infectious Mononucleosis

**DOI:** 10.7759/cureus.20395

**Published:** 2021-12-13

**Authors:** Sneha Adidam, Srikanth Adidam Venkata, Gregory Benn, Philip Oppong-Twene, Robert A Delapenha

**Affiliations:** 1 Internal Medicine, Howard University Hospital, Washington, D.C., USA; 2 Internal Medicine, Brookdale University Hospital Medical Center, New York, USA

**Keywords:** self-limiting, direct hyperbilirubinemia, liver injury biomarkers, hepatocellular injury, infectious mononucleosis

## Abstract

Hepatic dysfunction in the setting of infectious mononucleosis has been documented in the literature. However, clinically significant jaundice and direct hyperbilirubinemia are rarely associated with this infection. In the instance of undetermined underlying diagnosis and hepatic enzyme derangement, this may pose a diagnostic challenge. Furthermore, several diagnostic tests may be indicated, which could potentially increase resource consumption in any hospital setting. This case report aims to remind physicians that infectious mononucleosis may be a cause of hyperbilirubinemia, which does not usually require further complex testing other than monitoring and supportive therapy.

## Introduction

Infectious mononucleosis is a clinical syndrome usually diagnosed among young patients who typically present with symptoms such as fatigue, fever, and sore throat [1]. The diagnosis is made by testing for either heterophile antibodies or EBV-specific antibodies [2]. The virus is spread by the exchange of oral secretions and has an incubation period of roughly six weeks [3]. EBV infectious mononucleosis has been found to cause hepatic dysfunction. The pathologies seen in acute hepatitis include EBV-associated hemolytic anemia, cytotoxic liver injury, or cholestasis. Among adult cases described, 55% are in keeping with a cholestatic pattern. Typically, the derangement in liver enzymes is transient and self-limited [4,5]. We report herein the case of a 20-year-old undergraduate student with nonspecific symptoms including sore throat and dark urine with laboratory workup showing altered liver-associated enzymes.

## Case presentation

A 20-year-old African American female with no significant medical history presented to the ED with dark brown urine and a sore throat for approximately one week. She also endorsed fatigue and abdominal discomfort within the same one-week duration. She denied cough, shortness of breath, or urinary symptoms. Notably, the patient is an undergraduate student, and she lives in a college dorm. The patient is heterosexual with one male partner with whom she is sexually active. Her partner also experienced similar symptoms within the same period. She denied any use of medications or herbal supplements. She denied alcohol use, cigarette smoking, or recreational drug use. She also denied acquiring tattoos or piercings, a history of sexually transmitted infections, or previous liver disease and viral hepatitis. However, she gave a history of liver disease in her maternal aunt, without further details.

Vitals signs at presentation included a temperature of 100.4°F, blood pressure of 122/81 mmHg, heart rate of 82 beats per minute, oxygen saturation of 100% on room air, and respiratory rate of 18 breaths per minute. Physical examination was significant for scleral icterus, mild diffuse abdominal tenderness worsened by deep palpation, and a palpable liver edge 3 cm below the costal margin. The patient was fully alert and oriented, and no signs of encephalopathy were noted. There were no palpable lymph nodes.

Laboratory values are as seen in Table 1. Furthermore, electrolytes were within normal limits. Urinalysis was positive for bilirubin. Abdominal ultrasound showed prominence of both the liver and spleen. The common bile duct measured 0.29 cm with no evidence of ductal dilatation. Blood cultures were negative.

The patient was managed conservatively with intravenous fluids and antipyretics as needed. Liver enzymes showed a downtrend with improvement in clinical status. The patient was discharged with outpatient follow-up of liver function tests. The patient was also counseled to avoid contact sports.

**Table 1:** Significant laboratory investigations

Laboratory test	Value on admission	Reference range
Alkaline phosphatase	202 IU/L	30–130 IU/L
Alanine transaminase	436 IU/L	0–55 IU/L
Aspartate transaminase	399 IU/L	0–50 IU/L
Direct bilirubin	3.2 mg/dL	0–0.2 mg/dL
Total bilirubin	4.1 mg/dL	0.2–1.2 mg/dL
White blood cell count	15.55 × 10^9^	3.2–10.6 × 10^9^
Lymphocyte	71.1%	11%–49%
Antinuclear antibody	Negative	Negative
Anti-mitochondrial antibody	Negative	Negative
SARS-CoV-2 RNA (COVID- 19)	Negative	Negative
Human immunodeficiency virus	Negative	Negative
Mononucleosis test (heterophile antibody)	Positive	Negative
Hepatitis B surface antigen/antibody	Negative	Negative
Hepatitis A IgM antibody	Negative	Negative
Hepatitis C antibody	Negative	Negative

## Discussion

Young adults with infectious mononucleosis have been noted to have liver involvement in approximately 10% of cases [6]. An elevation in transaminase levels of 5-10 times the upper limit of normal with jaundice, as in our index case, occurs in <5% of cases of EBV infectious mononucleosis. It is more common to encounter mild and transient increases in transaminases. Hepatic failure due to EBV infection has sometimes contributed to mortality in such cases [7]. According to Patel et al., 17 cases were reported worldwide with 85% mortality [8].

Our patient’s liver function tests demonstrated a hepatocellular-cholestatic pattern of injury. The R factor was calculated to be 4.7, which indicates a mixed pattern. Biliary obstruction was ruled out by abdominal ultrasound, which showed no intrahepatic/extrahepatic duct obstruction or dilation. The hepatitis panel was negative, indicating that viral hepatitis due to hepatitis A, B, or C was less likely. Other more common causes include medication or herbal use; however, this patient denied using any medications. Cholestasis of pregnancy is unlikely given that the urinary pregnancy test was negative. It seems that the most likely cause after exclusion of other common causes was EBV infectious mononucleosis.

The mechanism by which EBV causes liver injury remains open to debate. An immune-mediated cause versus cytotoxic liver injury has been suggested [9]. There is also a mention of EBV inhibiting a bilirubin transporter called multidrug resistance protein 2 (MRP2) [10].

As in most cases of EBV infectious mononucleosis, this patient was managed with supportive treatment. There was no need to consider any further treatment such as steroids or antivirals. The patient showed rapid clinical and laboratory improvement and was discharged within 48 hours.

## Conclusions

It may be wise to have a high index of suspicion of EBV infectious mononucleosis in young patients presenting with a viral syndrome (fever, fatigue, and pharyngitis) in the setting of deranged liver function consistent with a hepatocellular-cholestatic pattern and no evidence of biliary obstruction. The learning point, in this case, is that the early diagnosis of EBV infectious mononucleosis is useful in selecting appropriate treatment and avoiding unnecessary, additional diagnostic and therapeutic measures.
